# Multimodal optical imaging of iris flocculi in three consecutive generations: a case report

**DOI:** 10.3389/fmed.2024.1369884

**Published:** 2024-08-29

**Authors:** Anna Jiang, Licong Liang, Kaiqin She, Fang Lu

**Affiliations:** Department of Ophthalmology, West China Hospital, Sichuan University, Chengdu, Sichuan, China

**Keywords:** iris flocculi, *ACTA2* gene, color photographs of the iris, optical coherence tomography (OCT), fundus angiography (FA), familial thoracic aortic aneurysm and aortic dissection (TAAD)

## Abstract

**Background:**

Multiple pigmented epithelial cysts at the edge of pupils, that is, iris flocculi, in both eyes, are rare ocular diseases. It has been demonstrated that this disease can be attributed to mutations in the smooth muscle α-actin 2 (*ACTA2*) gene, which mainly affects the function of smooth muscle cells (SMCs). SMCs are components of the iris, aorta, and several other systemic organs. In addition, iris flocculi are strongly correlated with familial thoracic aortic aneurysm and dissection (TAAD), which is caused by the mutation of amino acid 149 in the *ACTA2* gene.

**Case description:**

A 6-month-old Chinese boy was found to have iris flocculi during ocular fundus screening for premature infants. His mother, a 30-year-old Chinese woman with a history of aortic dissection, underwent an ophthalmic examination and was found to have iris flocculi. Whole exome sequencing revealed a heterozygous c.445C > T (p. Arg149Cys) mutation in *ACTA2* in both the boy and his mother. After his family history was traced, the boy’s grandfather was diagnosed with similar iris flocculi. Due to the absence of any ocular complications caused by iris flocculi in the cases, no special treatment was given, and regular follow-up was recommended.

**Conclusion:**

We reported one case of familial iris flocculi caused by a heterozygous missense mutation in *ACTA2* (p. Arg149Cys) and presented multimodal optical images of both the iris and fundus in three consecutive generations. This case report enriched the clinical features of retinal vasculature and macula associated with the mutation in the amino acid 149 of the *ACTA2* gene.

## Introduction

Primary iris cysts are sac-like structures that typically occur in the pigmented epithelium of the iris. They are composed primarily of pigment epithelium and goblet cells from the pigment cell layer of the iris (1). Cysts frequently manifest around or within the iris, with unilateral peripheral cysts being more prevalent. Cysts at the pupillary margin are relatively uncommon, representing approximately 3–13% of all primary pigment epithelial cysts (2). Shields reported multiple pigmented epithelial cysts at the pupillary margin as iris flocculi (1). Further investigations have indicated that iris flocculi may be attributed to a mutation in the smooth muscle α-2 actin (*ACTA2*) gene (3–5). This gene encodes the most abundant subtype of actin in smooth muscle cells (SMCs) in the iris, aorta, and multiple other vital organs (6). Mutations in the *ACTA2* gene can lead to malfunction of SMCs and often exhibit autosomal dominant inherited diseases. A number of studies have shown that iris flocculi are caused by a mutation in amino acid 149 of the *ACTA2* gene, which can simultaneously lead to familial thoracic aortic aneurysm and dissection (TAAD) (7–11). Consequently, the early detection of iris flocculi and the prompt diagnosis of aortic disease are of paramount importance. Other mutations in the *ACTA2* gene, such as the substitution at Arg179 (12) or Asn117 (13), have been reported to cause obvious tortuosity of retinal vessels, especially arteries. However, to the best of our knowledge, no one has described the clinical features of the retina in the mutation of amino acid 149. Herein, we present a familial case of iris flocculi resulting from the Arg149Cys mutation in *ACTA2* and describe the multimodal optical images of both the iris and retina in three consecutive generations.

Whole exome sequencing was used in this case; the exon is the protein-coding region of the human genome. Although the exon region constitutes less than 2% of the entire genome, it contains the disease-causing mutation sites of the majority of diseases (14). Consequently, whole exome sequencing represents a more cost-effective alternative to whole genome sequencing. It can enhance the sequencing depth, reduce the sequencing cycle, and thus facilitate the identification of low-frequency variants at a reduced cost.

## Case description

### Case 1

**Figure 1:**
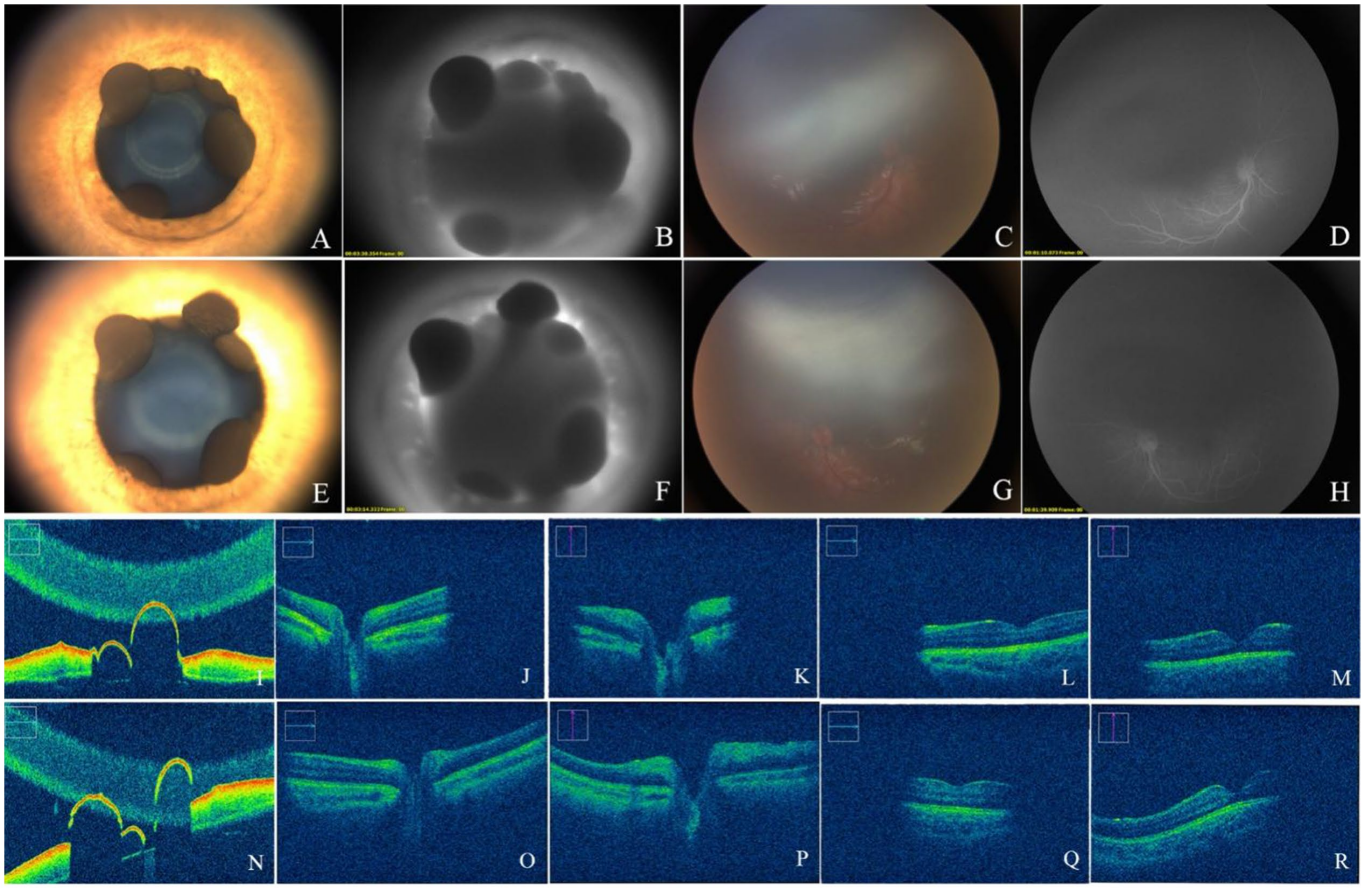
Photographs of the anterior segment showed multiple iris cysts at the pupillary margin of both eyes **(A,E)**. FA showed a slight leakage of fluorescein at the pupillary margin **(B,F)**. Fundus photographs showed a normal optic disk and macula **(C,G)**. FA showed slight tortuosity of the retinal arterioles, with no vascular leakage **(D,H)**. OCT showed that the cysts were hollow with a thin wall **(I,N)** a normal retinal optic disk **(J,K,O,P)**, and a macula **(L,M,Q,R)**.

**Figure 2:**
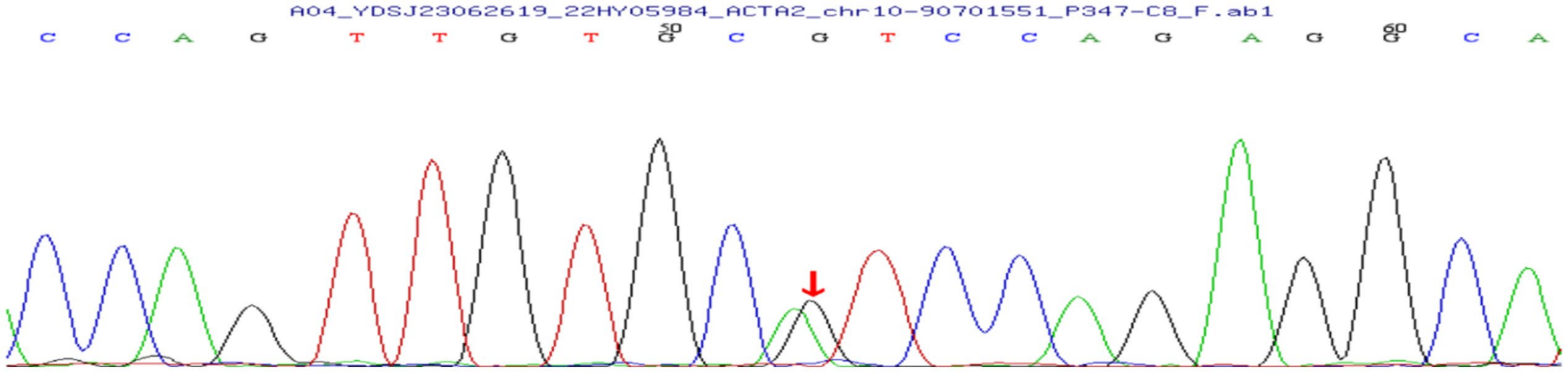
The whole exome genome sequencing showed a heterozygous c.445C > T mutation in ACTA2(the peak map shown by Sanger validation is the reverse complementary sequence of the detected bases).

A male infant was found to have iris flocculi by fundus screening for premature infants when he was 2 months old. The infant was born at 31^+6^ weeks of pregnancy, with a birth weight of 1720 grams. He had a history of invasive ventilation. At 6 months of age, he was admitted for ophthalmic examination under general anesthesia. Color photographs (RetCam III, Natus Medical, USA) of the iris showed multiple iris cysts with partial shrinkage at the pupillary margin in both eyes (Figures 1A,E). Iris angiography showed a slight leakage of fluorescein at the pupillary margin of both eyes, no fluorescent signals were detected within the iris flocculi (Figures 1B,F). At the same time, intraoperative optical coherence tomography (OCT) (OPMI LUMERA 700 and RESCAN 700, ZEISS) revealed multiple cysts with relatively smooth surface reflections at the pupillary margin (Figures 1I,N). OCT revealed normal morphology of the optic disk, macula, and fovea (Figures 1J–M,O). Because the pupils did not respond well to the mydriatics, fundus photographs (Figures 1C,G) and fluorescein angiography (FA) (Figures 1D,H) could not clearly display the entire retina. However, FA showed slight tortuosity of the retinal arterioles, with no detectable vascular leakage. Whole exome sequencing revealed a heterozygous c.445C > T (p. Arg149Cys) mutation in *ACTA2* (Figure 2). While his growth and development are within normal range, an ultrasonic cardiogram did not reveal cardiac or aortic abnormalities. Due to the absence of complications caused by iris flocculi in the patient, an outpatient follow-up for 3 months after discharge was recommended to perform an intraocular pressure examination and observe the changes in the iris flocculi. If there is no change in symptoms, the follow-up can be once a year.

### Case 2

**Figure 3:**
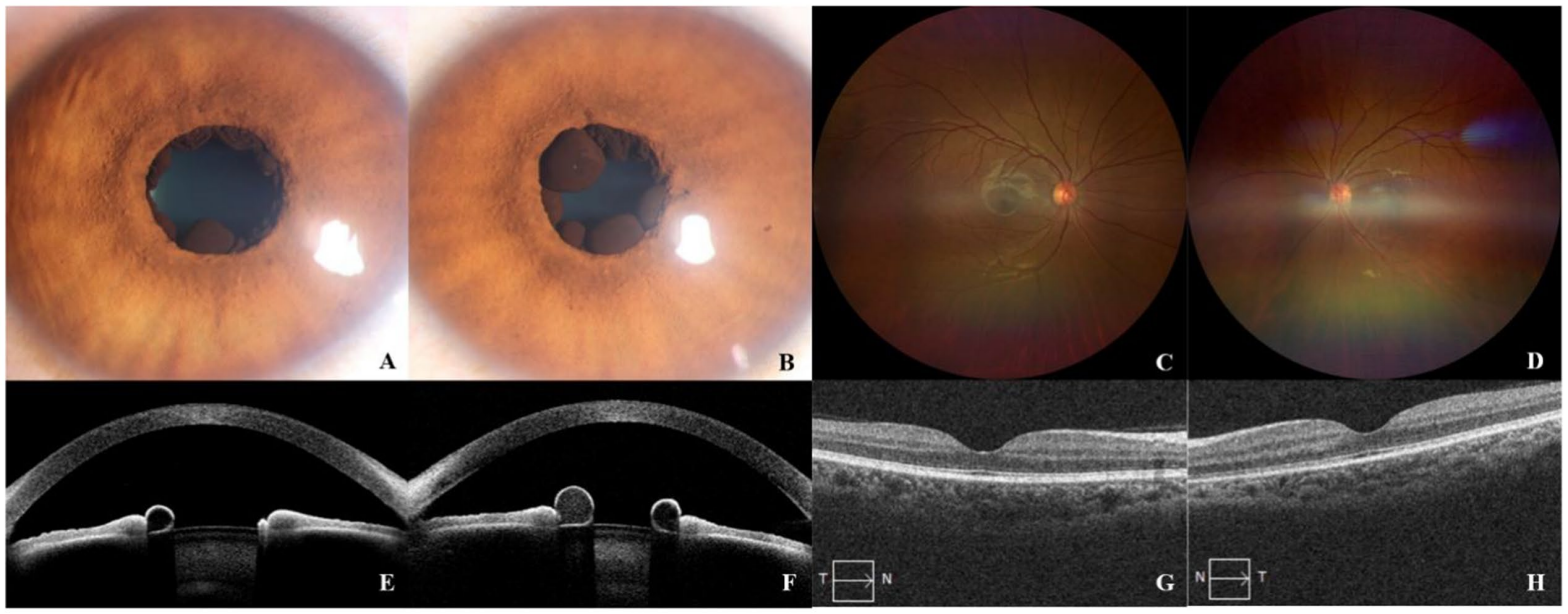
Color photographs showed multiple cysts at the pupillary margin, some showing partly collapsed with shrinking **(A,B)**. AS-OCT showed cystic components lining the pupillary margin of the iris **(E,F)**. Wide-angle color fundus photographs **(C,D)** and OCT **(G,H)** showed normal structure in the retinal vasculature and macula.

The mother of case 1, a 30-year-old woman, developed hypertension with chest and back pain during her pregnancy. She was diagnosed with aortic dissection and underwent intraluminal covered stent repair for aortic dissection. After being advised to undergo an ophthalmic examination, color photographs of the iris revealed multiple cysts at the pupillary margin in both eyes, and the iris crypts appeared to be less prominent (Figures 3A,B). A cystic, dark area of the iris flocculi was shown on the anterior segment OCT (AS-OCT). Some of the cysts exhibited different reflectivities, they may contain different components, and other cysts were thin-walled and hollow (Figures 3E,F). Wide-angle color fundus photographs (Clarus 500, Zeiss) (Figures 3C,D) and OCT (Figures 3G,H) did not reveal abnormalities in the retinal vasculature or macula. Genetic testing revealed the same mutation in *ACTA2* (p. Arg149Cys) as in case 1. Since there was no evidence of pupillary occlusion, corneal opacity, ocular inflammation, or elevated intraocular pressure, the patient was recommended to be followed up in the ophthalmic clinic every 6 months.

### Case 3

**Figure 4:**
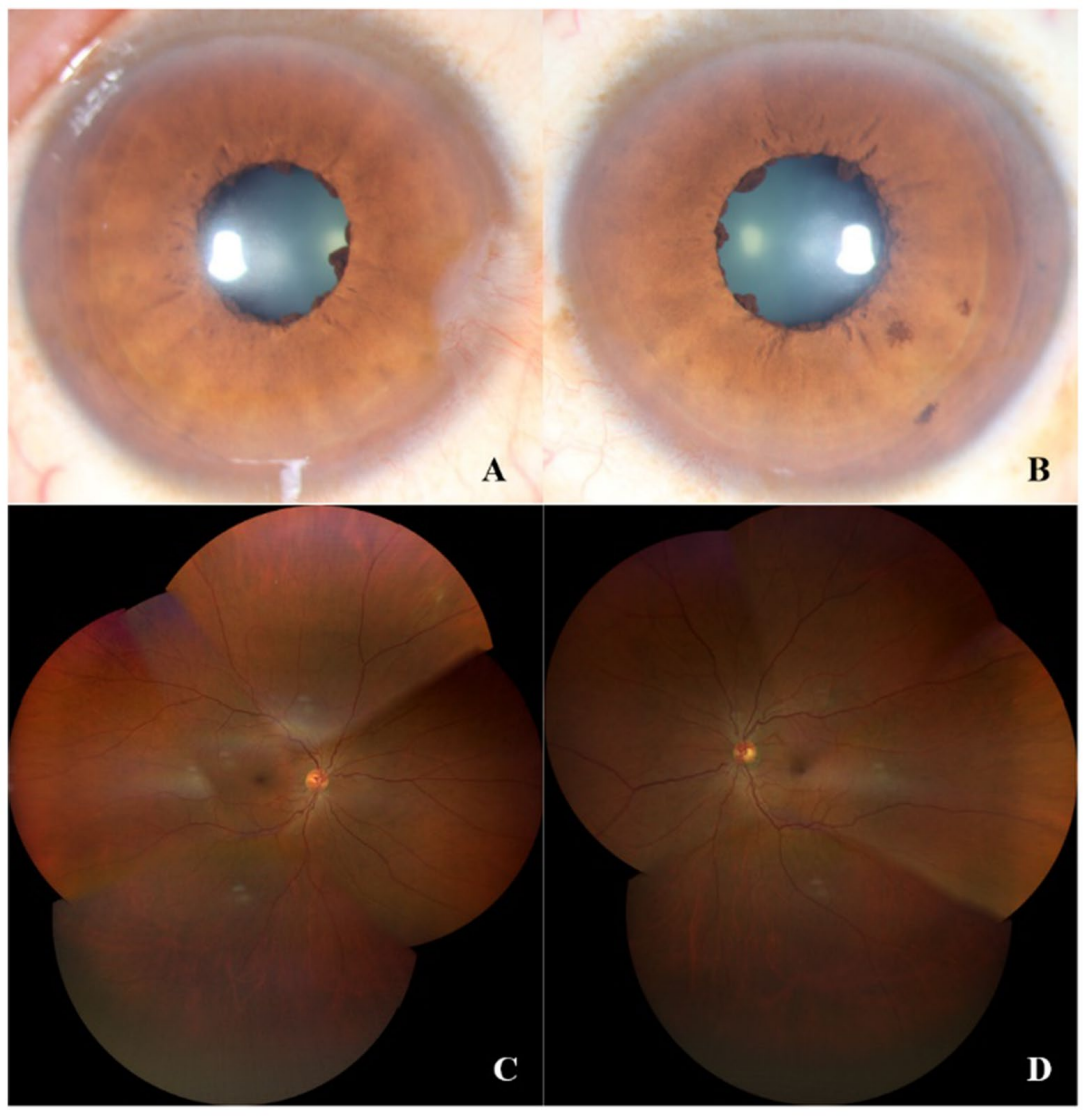
Color photographs showed iris flocculi in both eyes **(A,B)**. A montage of retinal photographs showed tortuous retinal small blood vessels **(C,D)**.

A 56-year-old man, the maternal grandfather of case 1, was examined via color photography, which revealed iris flocculi and obscure iris crypts in both eyes (Figures 4A,B). Most of the cysts were collapsed, forming wrinkled lesions. A montage of retinal photographs showed tortuous small retinal blood vessels (Figures 4C,D). The optic disk and macula were normal in both eyes. The intraocular pressure was within normal range, with no other symptomatic ocular comorbidities. The patient has no history of aortic disease and underwent aortic screening at a local hospital without any findings.

## Discussion

Multiple cysts of the bilateral iris are a rare hereditary condition. To our knowledge, this is the first reported case of iris flocculi in Chinese patients caused by Arg149 mutation in the *ACTA2* gene, and it is autosomal dominant in this family. During the process of fundus examination under general anesthesia, the child’s weak response to mydriatic drugs made it difficult to dilate the pupils, resulting in a limited image range and poor imaging of the fundus examination. Non-responsiveness to light and mydriatics has also been reported in other cases of Arg179 or Asn117 mutations with congenital pupillary dilation (15). An article describing the Asn117 mutation in *ACTA2* suggests that a hyporeflective band in front of the pigment epithelium on OCT of the iris indicates the presence of a malfunctioning sphincter (13). Therefore, it was speculated that dysfunction of the iris sphincter could be caused by the abnormality in the *ACTA2* gene, resulting in the limited pupillary response to mydriatics. The presence of persistent pupillary membrane, incomplete vascular degeneration, and vascular development at the posterior surface of the iris (16) could cause fluorescein leakage at the pupillary margin.

For the first time, we described the clinical features of the retina and macula through multimodal optical imaging in the case of the Arg149 mutation in *ACTA2*. Although the pupils could not be dilated sufficiently, which affected the observation of the retina, a normal macular foveal morphology could be observed by fundus color photography and OCT. A slight tortuosity of the retinal arterioles was revealed by fluorescein angiography. Given the child’s history of preterm birth and invasive ventilation, it is plausible that the abnormality may be related to this factor, However, no vascular features of retinopathy of prematurity (ROP) were identified via FA. Additionally, the child’s grandfather also exhibited an intermediate degree of vessel tortuosity in the retinal vasculature. Although other mutations in *ACTA2*, such as substitution at Arg179 or Asn117, have been reported in patients with retinal arteriolar tortuosity (12, 13), there is no such report in cases with an Arg149 mutation. Nevertheless, we have not yet identified the cause of abnormal retinal vasculature in either the child or his grandfather, and we cannot determine whether it was attributed to the mutation of *ACTA2.* We also found that iris crypts were less prominent in all eyes, which has been reported in cases with other *ACTA2* mutations (Arg179 or Asn117) in the past (17), while iris atrophy and depigmentation were more common in Arg149 mutation (13), and less prominent crypts were rarely reported. Despite the fact that *ACTA2* mutation impairs smooth muscle function, the pathophysiology of iris flocculi lesions associated with the mutation is yet unknown. Further research is therefore required to investigate the pathology of the iris flocculi. This is the first report of retinal abnormalities associated with the Arg149 mutation in *ACTA2*. However, further elaboration on this phenomenon is needed through long-term observation and the inclusion of additional cases. Although iris cysts are typically associated with glaucoma, our findings did not indicate the presence of such lesions in the child or mother and grandfather.

It is worth noting that the mother of the child developed an aortic dissection at the age of 30, while the child has not been detected with any aortic lesions. Previous case reports indicated that the occurrence of thoracic aortic aneurysm or aortic dissection caused by mutations in the *ACTA2* gene was age-related. In most cases, patients had a lower probability of developing aortic lesions in childhood, but these lesions can occur in adulthood (18). This indicates that although aortic disease has been excluded in the child, continued monitoring and related assessments are necessary. The patient needs to be vigilant and promptly treated to prevent life-threatening complications.

## Conclusion

In conclusion, we identified and reported a familial case of iris flocculi in three consecutive generations. Whole exome sequencing revealed mutations in the *ACTA2* (p. Arg149Cys) gene, and multimodal imaging technology revealed the characteristics of ocular lesions in this family. The mother of the proband developed aortic dissection lesions, confirming the strong correlation between iris flocculi, familial TAAD, and the *ACTA2* (p. Arg149Cys) mutation, which also assists in the early detection of aortic lesions in clinical practice. Through FA, abnormalities in the retinal vasculature were discovered. The present report offers further clinical insights and avenues for investigation into the ocular lesions caused by mutations in *ACTA2*.

## Data Availability

The original contributions presented in the study are included in the article/Supplementary material, further inquiries can be directed to the corresponding author.
