# Supplementary material for: Multimodal optical imaging of iris flocculi in three consecutive generations: a case report
Source: Front Med (Lausanne). 2024 Aug 29;11:1369884. doi: 10.3389/fmed.2024.1369884 (PMC11390461; doi:10.3389/fmed.2024.1369884)
Supplement: Supplementary file 1 [file Table_1.pdf]

CARE case report guidelines

CARE Checklist of information to include when writing a case report

(cc) BY-NC-ND

Topic	Item	Checklist item description	Reported on Line
Title	1	The diagnosis or intervention of primary focus followed by the words "case report"	line 55-59
Key Words	2	2 to 5 key words that identify diagnoses or interventions in this case report, including "case report"	line 93-95
Abstract (no references)	3a	Introduction: What is unique about this case and what does it add to the scientific literature?	line 84-90
	3b	Main symptoms and/or important clinical findings	line 75-80
	3c	The main diagnoses, therapeutic interventions, and outcomes	line 80-83
	3d	Conclusion—What is the main "take-away" lesson(s) from this case?	line 84-90
Introduction	4	One or two paragraphs summarizing why this case is unique (**may include references**)	line 102-124
Patient Information	5a	De-identified patient specific information.	line 139-141,176,198
	5b	Primary concerns and symptoms of the patient.	139-140,177-181,199
	5c	Medical, family, and psycho-social history including relevant genetic information	line 157-159
	5d	Relevant past interventions with outcomes	NA
Clinical Findings	6	Describe significant physical examination (PE) and important clinical findings.	143-159,180-189,199-202
Timeline	7	Historical and current information from this episode of care organized as a timeline	NA
Diagnostic Assessment	8a	Diagnostic testing (such as PE, laboratory testing, imaging, surveys).	143-159,180-189,199-202
	8b	Diagnostic challenges (such as access to testing, financial, or cultural)	line 215-216
	8c	Diagnosis (including other diagnoses considered)	line 139,181,199
	8d	Prognosis (such as staging in oncology) where applicable	NA
Therapeutic Intervention	9a	Types of therapeutic intervention (such as pharmacologic, surgical, preventive, self-care)	line 167-171
	9b	Administration of therapeutic intervention (such as dosage, strength, duration)	NA
	9c	Changes in therapeutic intervention (with rationale)	NA
Follow-up and Outcomes	10a	Clinician and patient-assessed outcomes (if available)	NA
	10b	Important follow-up diagnostic and other test results	NA
	10c	Intervention adherence and tolerability (How was this assessed?)	NA
	10d	Adverse and unanticipated events	NA
Discussion	11a	A scientific discussion of the strengths AND limitations associated with this case report	212,277-282,396-422
	11b	Discussion of the relevant medical literature **with references**.	220-276,336-340,389-396
	11c	The scientific rationale for any conclusions (including assessment of possible causes)	line 427-431,220-223
	11d	The primary "take-away" lessons of this case report (without references) in a one paragraph conclusion	line 439-449
Patient Perspective	12	The patient should share their perspective in one to two paragraphs on the treatment(s) they received	NA
Informed Consent	13	Did the patient give informed consent? Please provide if requested	Yes √ No ☐
